# Supplementary material for: End Sequence Analysis Toolkit (ESAT) expands the extractable information from single-cell RNA-seq data
Source: Genome Res. 2016 Oct;26(10):1397–410. doi: 10.1101/gr.207902.116 (PMC5052061; doi:10.1101/gr.207902.116)
Supplement: Supplemental Material [file supp_gr.207902.116_Supp_Materials.tar.gz › ESAT-esat_v0.1/doc/html/classbroad_1_1pda_1_1datastructures_1_1_location_aware_matrix-members.html]

ESAT: Member List


ESAT


- Main Page
- Related Pages
- Packages
- Classes
- Files

- Class List
- Class Index
- Class Hierarchy
- Class Members

All Classes Namespaces Files Functions Variables Enumerator Pages

- broad
- pda
- datastructures
- LocationAwareMatrix

broad.pda.datastructures.LocationAwareMatrix Member List

This is the complete list of members for broad.pda.datastructures.LocationAwareMatrix, including all inherited members.

|  |  |  |
| --- | --- | --- |
| add(MatrixWithHeaders m2) | broad.core.datastructures.MatrixWithHeaders |  |
| addColumn(String column) | broad.core.datastructures.MatrixWithHeaders |  |
| addRow(String row, String description, double[] vals) | broad.core.datastructures.MatrixWithHeaders |  |
| annotation(GenomicAnnotation annotation) | broad.pda.datastructures.LocationAwareMatrix |  |
| append(MatrixWithHeaders other) | broad.core.datastructures.MatrixWithHeaders |  |
| appendColumns(MatrixWithHeaders other) | broad.core.datastructures.MatrixWithHeaders |  |
| appendRows(LocationAwareMatrix other) | broad.pda.datastructures.LocationAwareMatrix |  |
| broad::core::datastructures::MatrixWithHeaders.appendRows(MatrixWithHeaders other) | broad.core.datastructures.MatrixWithHeaders |  |
| begin() | broad.pda.datastructures.LocationAwareMatrix |  |
| browserLine(String line) | broad.pda.datastructures.LocationAwareMatrix |  |
| columnDensityNormalization() | broad.core.datastructures.MatrixWithHeaders |  |
| columnDimension() | broad.core.datastructures.MatrixWithHeaders |  |
| compressMatrixByRowDescriptions() | broad.core.datastructures.MatrixWithHeaders |  |
| confidenceByColumn(List< String > controlRows) | broad.core.datastructures.MatrixWithHeaders |  |
| containsColumn(String colName) | broad.core.datastructures.MatrixWithHeaders |  |
| containsRow(String rowName) | broad.core.datastructures.MatrixWithHeaders |  |
| copy() | broad.core.datastructures.MatrixWithHeaders |  |
| dataInverse() | broad.core.datastructures.MatrixWithHeaders |  |
| eof() | broad.pda.datastructures.LocationAwareMatrix |  |
| excludeByRowNames(Collection< String > flaggedGenes) | broad.core.datastructures.MatrixWithHeaders |  |
| filterInvariantGenes(MatrixWithHeaders data, double fold) | broad.core.datastructures.MatrixWithHeaders |  |
| filterValuesLargerThanUsingColumn(int colIdx, double maxValue) | broad.core.datastructures.MatrixWithHeaders |  |
| filterValuesRangeUsingColumn(int colIdx, double minVal, double maxVal) | broad.core.datastructures.MatrixWithHeaders |  |
| filterValuesSmallerThanUsingColumn(int colIdx, double minValue) | broad.core.datastructures.MatrixWithHeaders |  |
| floor(double floor) | broad.core.datastructures.MatrixWithHeaders |  |
| get(int i, int j) | broad.core.datastructures.MatrixWithHeaders |  |
| get(String row, String column) | broad.core.datastructures.MatrixWithHeaders |  |
| get(String rowName, int colIdx) | broad.core.datastructures.MatrixWithHeaders |  |
| get(int rowIdx, String colName) | broad.core.datastructures.MatrixWithHeaders |  |
| getChromosomes() | broad.pda.datastructures.LocationAwareMatrix |  |
| getClosest(LightweightGenomicAnnotation annotation) | broad.pda.datastructures.LocationAwareMatrix |  |
| getColoumnName(int i) | broad.core.datastructures.MatrixWithHeaders |  |
| getColumn(int j) | broad.core.datastructures.MatrixWithHeaders |  |
| getColumn(String columnName) | broad.core.datastructures.MatrixWithHeaders |  |
| getColumnNames() | broad.core.datastructures.MatrixWithHeaders |  |
| getData() | broad.core.datastructures.MatrixWithHeaders |  |
| getIndecesForRowDescription(String rowDescription) | broad.core.datastructures.MatrixWithHeaders |  |
| getMeanOverAllRows() | broad.core.datastructures.MatrixWithHeaders |  |
| getMedianOverAllRows() | broad.core.datastructures.MatrixWithHeaders |  |
| getNanostringProbeClasses() | broad.core.datastructures.MatrixWithHeaders |  |
| getNanostringReplicateMap() | broad.core.datastructures.MatrixWithHeaders |  |
| getNextClosest(LightweightGenomicAnnotation annotation) | broad.pda.datastructures.LocationAwareMatrix |  |
| getNumberColumns() | broad.core.datastructures.MatrixWithHeaders |  |
| getNumberRows() | broad.core.datastructures.MatrixWithHeaders |  |
| getOverlappers(LightweightGenomicAnnotation annotation) | broad.pda.datastructures.LocationAwareMatrix |  |
| getPIDToName() | broad.core.datastructures.MatrixWithHeaders |  |
| getPriorClosest(LightweightGenomicAnnotation annotation) | broad.pda.datastructures.LocationAwareMatrix |  |
| getRow(String rowName) | broad.core.datastructures.MatrixWithHeaders |  |
| getRow(int i) | broad.core.datastructures.MatrixWithHeaders |  |
| getRow(String gene, Collection< String > group1) | broad.core.datastructures.MatrixWithHeaders |  |
| getRowAnnotations() | broad.pda.datastructures.LocationAwareMatrix |  |
| getRowDescriptions() | broad.core.datastructures.MatrixWithHeaders |  |
| getRowLocationMap() | broad.pda.datastructures.LocationAwareMatrix |  |
| getRowName(int i) | broad.core.datastructures.MatrixWithHeaders |  |
| getRowNames() | broad.core.datastructures.MatrixWithHeaders |  |
| getRowPosition(String row) | broad.pda.datastructures.LocationAwareMatrix |  |
| getRowTree(String chromosome) | broad.pda.datastructures.LocationAwareMatrix |  |
| getValues(String gene, Collection< String > controls) | broad.core.datastructures.MatrixWithHeaders |  |
| hasColumn(String column) | broad.core.datastructures.MatrixWithHeaders |  |
| hasNanostringProbeClasses() | broad.core.datastructures.MatrixWithHeaders |  |
| hasRow(String row) | broad.core.datastructures.MatrixWithHeaders |  |
| initColIndexMaps(List< String > columnNameList) | broad.core.datastructures.MatrixWithHeaders | protected |
| initFromIGVMatrix(BufferedReader br, String header, String chromosomeToLoad) | broad.pda.datastructures.LocationAwareMatrix | protected |
| initFromNanostring(BufferedReader br, String header) | broad.core.datastructures.MatrixWithHeaders | protected |
| initFromReader(BufferedReader br) | broad.core.datastructures.MatrixWithHeaders | protected |
| initFromRegularMatrix(BufferedReader br, String header) | broad.core.datastructures.MatrixWithHeaders | protected |
| initGCTFromReader(BufferedReader br) | broad.core.datastructures.MatrixWithHeaders | protected |
| initNameIndexMaps(List< String > rowNameList, List< String > columnNameList) | broad.core.datastructures.MatrixWithHeaders | protected |
| initRowDescrIndexMaps(List< String > rowDescriptionList) | broad.core.datastructures.MatrixWithHeaders | protected |
| initRowIndexMaps(List< String > rowNameList) | broad.core.datastructures.MatrixWithHeaders | protected |
| isColumnNonTrivial(String colName) | broad.core.datastructures.MatrixWithHeaders |  |
| LocationAwareMatrix() | broad.pda.datastructures.LocationAwareMatrix |  |
| LocationAwareMatrix(List< String > rows, List< String > columns, Map< String, LightweightGenomicAnnotation > rowLocations) | broad.pda.datastructures.LocationAwareMatrix |  |
| LocationAwareMatrix(BufferedReader br, String chr) | broad.pda.datastructures.LocationAwareMatrix |  |
| LocationAwareMatrix(List<?extends LightweightGenomicAnnotation > probeData, List< String > samples) | broad.pda.datastructures.LocationAwareMatrix |  |
| LocationAwareMatrix(MatrixWithHeaders mwh) | broad.pda.datastructures.LocationAwareMatrix |  |
| log10() | broad.core.datastructures.MatrixWithHeaders |  |
| log10(double fudge) | broad.core.datastructures.MatrixWithHeaders |  |
| log2() | broad.core.datastructures.MatrixWithHeaders |  |
| log2(double fudge) | broad.core.datastructures.MatrixWithHeaders |  |
| MatrixWithHeaders() | broad.core.datastructures.MatrixWithHeaders | protected |
| MatrixWithHeaders(String inputFile) | broad.core.datastructures.MatrixWithHeaders |  |
| MatrixWithHeaders(BufferedReader br) | broad.core.datastructures.MatrixWithHeaders |  |
| MatrixWithHeaders(Matrix data, List< String > rowNames, List< String > colNames) | broad.core.datastructures.MatrixWithHeaders |  |
| MatrixWithHeaders(Matrix data, List< String > rowNames, List< String > colNames, List< String > rowDescriptions) | broad.core.datastructures.MatrixWithHeaders |  |
| MatrixWithHeaders(List< String > rows, List< String > columns, Map< String, String > pidToName) | broad.core.datastructures.MatrixWithHeaders |  |
| MatrixWithHeaders(MatrixWithHeaders matrix, List< String > rows, List< String > columns) | broad.core.datastructures.MatrixWithHeaders |  |
| MatrixWithHeaders(List< String > rows, List< String > columns) | broad.core.datastructures.MatrixWithHeaders |  |
| MatrixWithHeaders(List< String > rows, List< String > columns, List< String > rowDescriptions) | broad.core.datastructures.MatrixWithHeaders |  |
| MatrixWithHeaders(String gctFile, String chipFile) | broad.core.datastructures.MatrixWithHeaders |  |
| MatrixWithHeaders(List< List< Double >> rawData, ArrayList< String > rowNameList, List< String > columnNameList) | broad.core.datastructures.MatrixWithHeaders |  |
| MatrixWithHeaders(ArrayList< String > rows, ArrayList< String > columns, int DefMatVal) | broad.core.datastructures.MatrixWithHeaders |  |
| meanNormalizeColumns() | broad.core.datastructures.MatrixWithHeaders |  |
| meanNormalizeRows() | broad.core.datastructures.MatrixWithHeaders |  |
| medianCenterColumns() | broad.core.datastructures.MatrixWithHeaders |  |
| medianCenterColumnsThis() | broad.core.datastructures.MatrixWithHeaders |  |
| medianCenterRows() | broad.core.datastructures.MatrixWithHeaders |  |
| medianCenterRowsThis() | broad.core.datastructures.MatrixWithHeaders |  |
| medianNormalizeColumns() | broad.core.datastructures.MatrixWithHeaders |  |
| medianNormalizeRows() | broad.core.datastructures.MatrixWithHeaders |  |
| minus(MatrixWithHeaders other) | broad.core.datastructures.MatrixWithHeaders |  |
| multiplyColumnsWithConstants(double[] constants) | broad.core.datastructures.MatrixWithHeaders |  |
| multiplyColumnsWithConstants(Map< String, Double > constants) | broad.core.datastructures.MatrixWithHeaders |  |
| normalizeBy(double constant) | broad.core.datastructures.MatrixWithHeaders |  |
| normalizeByMean() | broad.core.datastructures.MatrixWithHeaders |  |
| normalizeColumnsByMean() | broad.core.datastructures.MatrixWithHeaders |  |
| normalizeColumnsByMedian() | broad.core.datastructures.MatrixWithHeaders |  |
| pow() | broad.core.datastructures.MatrixWithHeaders |  |
| quantileNormalize() | broad.core.datastructures.MatrixWithHeaders |  |
| quantileNormalizeColumns() | broad.core.datastructures.MatrixWithHeaders |  |
| randomPermuteColumns() | broad.core.datastructures.MatrixWithHeaders |  |
| rank() | broad.core.datastructures.MatrixWithHeaders |  |
| removeOverlappingRows(LocationAwareMatrix other) | broad.pda.datastructures.LocationAwareMatrix |  |
| round() | broad.core.datastructures.MatrixWithHeaders |  |
| rowDensityNormalization() | broad.core.datastructures.MatrixWithHeaders |  |
| rowDimension() | broad.core.datastructures.MatrixWithHeaders |  |
| scaleNorm() | broad.core.datastructures.MatrixWithHeaders |  |
| set(int i, int j, double val) | broad.core.datastructures.MatrixWithHeaders |  |
| set(String row, String column, double value) | broad.core.datastructures.MatrixWithHeaders |  |
| set(String row, int colIdx, double value) | broad.core.datastructures.MatrixWithHeaders |  |
| set(int rowIdx, String column, double value) | broad.core.datastructures.MatrixWithHeaders |  |
| setColumn(double[] vals, String column) | broad.core.datastructures.MatrixWithHeaders |  |
| setColumn(double[] vals, int column) | broad.core.datastructures.MatrixWithHeaders |  |
| setColumns(List< String > columns) | broad.pda.datastructures.LocationAwareMatrix |  |
| setData(Matrix data) | broad.core.datastructures.MatrixWithHeaders | protected |
| setDataDimensions(int rowDim, int columnDim) | broad.pda.datastructures.LocationAwareMatrix |  |
| setPIDToName(Map< String, String > rowDescriptions) | broad.core.datastructures.MatrixWithHeaders |  |
| setRow(String row, double[] vals) | broad.core.datastructures.MatrixWithHeaders |  |
| setRowDescription(int rowIdx, String rowDescription) | broad.core.datastructures.MatrixWithHeaders |  |
| setRowDescription(String row, String rowDescription) | broad.core.datastructures.MatrixWithHeaders |  |
| sortList(int columnIndex) | broad.core.datastructures.MatrixWithHeaders |  |
| submatrixByColumnIndex(Collection< Integer > columnIndex) | broad.core.datastructures.MatrixWithHeaders |  |
| submatrixByColumnNames(Collection< String > columnNames) | broad.core.datastructures.MatrixWithHeaders |  |
| submatrixByColumnNames(String[] columnNames) | broad.core.datastructures.MatrixWithHeaders |  |
| submatrixByColumns(Collection< String > columnNames) | broad.pda.datastructures.LocationAwareMatrix |  |
| submatrixByColumns(String[] columnNames) | broad.pda.datastructures.LocationAwareMatrix |  |
| submatrixByRowDescriptions(Collection< String > rowDescriptions, List< String > newRowNames) | broad.core.datastructures.MatrixWithHeaders |  |
| submatrixByRowNames(Collection< String > rowNames) | broad.core.datastructures.MatrixWithHeaders |  |
| submatrixByRowNames(String rowName) | broad.core.datastructures.MatrixWithHeaders |  |
| submatrixByRowNames(String[] rowNames) | broad.core.datastructures.MatrixWithHeaders |  |
| times(MatrixWithHeaders other) | broad.core.datastructures.MatrixWithHeaders |  |
| toMap() | broad.core.datastructures.MatrixWithHeaders |  |
| track(String line) | broad.pda.datastructures.LocationAwareMatrix |  |
| tScoresByRow(List< Integer > controlRows, List< Integer > sampleRows, String comparisonName, double fudgeFactor) | broad.core.datastructures.MatrixWithHeaders |  |
| write(BufferedWriter bw) | broad.pda.datastructures.LocationAwareMatrix |  |
| broad::core::datastructures::MatrixWithHeaders.write(String fileName) | broad.core.datastructures.MatrixWithHeaders |  |
| writeBox(String save) | broad.core.datastructures.MatrixWithHeaders |  |
| writeCLS(String string, Collection< String > subset, Collection< String > negatives) | broad.core.datastructures.MatrixWithHeaders |  |
| writeColoumnNamesToFile(String outFile) | broad.core.datastructures.MatrixWithHeaders |  |
| writeGCT(BufferedWriter bw) | broad.core.datastructures.MatrixWithHeaders |  |
| writeGCT(String save) | broad.core.datastructures.MatrixWithHeaders |  |
| writeGCT(String save, Collection< String > columns) | broad.core.datastructures.MatrixWithHeaders |  |
| writeGCT(String save, Map< String, Collection< String >> experimentInfo, boolean ordered) | broad.core.datastructures.MatrixWithHeaders |  |
| writeGCT(String save, Map< String, Collection< String >> experimentInfo, Map< String, ExpressionExperimentInfo > fullInfo, boolean info) | broad.core.datastructures.MatrixWithHeaders |  |
| writeGCT(String save, Collection< String > columns, Collection< String > rows) | broad.core.datastructures.MatrixWithHeaders |  |
| writeGCT(String save, Collection< String > columns, Collection< String > rows, int precision) | broad.core.datastructures.MatrixWithHeaders |  |
| writeGCT(String save, Map< String, String > columns, Map< String, String > rows) | broad.core.datastructures.MatrixWithHeaders |  |
| writeGCT(String save, Map< String, String > columns, Collection< String > rows) | broad.core.datastructures.MatrixWithHeaders |  |
| writeGCT(String save, Map< String, String > columns) | broad.core.datastructures.MatrixWithHeaders |  |
| writeGCT(String save, File chipFile) | broad.core.datastructures.MatrixWithHeaders |  |
| writeGCTAndCLS(String save, Map< String, ExpressionExperimentInfo > experimentInfo) | broad.core.datastructures.MatrixWithHeaders |  |
| writeGCTWithHeaders(String save, Map< String, String > columnMapping) | broad.core.datastructures.MatrixWithHeaders |  |
| writeGMT(String save, double fold) | broad.core.datastructures.MatrixWithHeaders |  |
| writeRowNamesToFile(String outFile) | broad.core.datastructures.MatrixWithHeaders |  |
| zColumnNormalize() | broad.core.datastructures.MatrixWithHeaders |  |
| zRowNormalize() | broad.core.datastructures.MatrixWithHeaders |  |
| zScoresByColumn(List< String > controlRows) | broad.core.datastructures.MatrixWithHeaders |  |


---

Generated on Fri Aug 15 2014 15:10:59 for ESAT by  

 1.8.7
